# Financial challenges of being on long-term, high-cost medications

**DOI:** 10.1093/nop/npae098

**Published:** 2024-12-03

**Authors:** Cleopatra Elshiekh, Roberta Rudà, Edward R Scheffer Cliff, Francesca Gany, Joshua A Budhu

**Affiliations:** Immigrant Health and Cancer Disparities Service, Department of Psychiatry, Memorial Sloan Kettering Cancer Center, New York, New York, USA; Division of Neuro-Oncology, Department of Neuroscience, University and City of Health and Science Hospital, Turin, Italy; Program On Regulation, Therapeutics, And Law, Brigham and Women’s Hospital, Harvard Medical School, Boston, Massachusetts, USA; Immigrant Health and Cancer Disparities Service, Department of Psychiatry, Memorial Sloan Kettering Cancer Center, New York, New York, USA; Department of Neurology, Memorial Sloan Kettering Cancer Center, New York, New York, USA

**Keywords:** cost-effectiveness analysis, drug pricing, financial toxicity, health equity, social determinants of health

## Abstract

The isocitrate dehydrogenase (IDH) inhibitor, vorasidenib, may offer a promising new treatment option for patients with IDH-mutant gliomas. However, the indefinite nature of this targeted therapy raises significant financial concerns. High costs of targeted cancer therapies, often exceeding $150 000 annually, contribute to financial toxicity, characterized by medical debt, income loss, and psychological stress, and place stress on health systems. This review analyzes the drug approval and pricing mechanisms in various countries and their impact on healthcare costs and patient access, focusing specifically on the impacts in neuro-oncology. The United States employs a market-driven approach resulting in higher drug prices, while most countries, such as the United Kingdom, Germany, France, Italy, Japan, South Africa, and Brazil, use negotiated pricing and health technology assessment to manage costs. The financial burden of expensive medications affects patient adherence and quality of life, with many cancer patients facing substantial out-of-pocket expenses and potential treatment abandonment, and many more unable to access these drugs altogether. Vorasidenib’s introduction, while potentially improving patient outcomes, may exacerbate financial toxicity unless mitigated by patient access programs and cost-management strategies. As neuro-oncology treatment paradigms evolve, understanding the economic implications of new therapies is essential to ensure equitable access and optimize patient care.

**Key Points**

- Financial toxicity is a pressing concern for patients with brain tumors.
- New targeted therapies for glioma may increase overall healthcare spending for both patients and payers.

The advent of the isocitrate dehydrogenase (IDH) inhibitor, vorasidenib, in glioma represents exciting progress for patients with IDH-mutant gliomas and neuro-oncology clinicians.^1,2^ An oral, targeted therapy represents a new model for neuro-oncology; many current treatments have finite cycles with expected end dates. The optimal timing and duration of treatment with targeted therapies are less clear, with some patients continuing them indefinitely. Patients with IDH-mutant gliomas might be treated with IDH inhibitors for years if the medication continues to keep the tumor from progressing.

The use of indefinite therapies has profound financial implications for patients and the healthcare system. Targeted therapies in other cancer types have yielded substantial increases in spending.^3^ These medicines typically cost upwards of $150 000 USD per year and can contribute to financial toxicity, which describes the financial side effects imposed by treatment, such as medical debt, loss of income, and psychological stress.^4,5^ This review will provide an overview of the drug approval process and drug pricing in a few specified countries, as well as an overview of the costs of long-term medications. These include the financial implications that accompany long-term medications, including financial toxicity. Our field must be conscious of these implications, as these costs can and will be passed on to our patients and place strain on health systems and taxpayers.

## Cost of Healthcare and Medications

Healthcare costs have increased substantially over the past 40 years, in some cases doubling as a percentage of a country’s gross domestic product (GDP).^6^ For example, in 1980, the United States spent 8.2% of its GDP on healthcare; in 2021, that percentage had increased to 17.8%. Adjusted for inflation, the absolute dollar amount has more than doubled, with more than $4.5 trillion USD (all monetary amounts hereafter are given in US Dollars, unless otherwise specified) spent on healthcare in the United States in 2022.^6^ One important component of this increase in healthcare spending is prescription drugs, with global spending topping $1.3 trillion worldwide.^7^

The increase in drug prices is multifactorial. Although research and development costs represent one contribution to increasing prices, the profit motive of pharmaceutical companies combined with the lack of price regulation, especially in the United States, is a large driver of rapidly increasing prices.^8,9^ Large pharmaceutical companies in the United States have reported higher profits compared to non-pharmaceutical companies in the S&P 500 over the past 20 years.^8^ Cancer drugs in particular have been extremely profitable for pharmaceutical companies, with one study showing an average of $14.50 return on investment for every $1 spent on research and development.^10^

Novel treatment modalities, such as chimeric antigen receptor (CAR) T-cell therapy, offer new treatment options for patients with lymphoma, leukemia, and multiple myeloma. However, these treatments can cost up to $1 million per patient, limiting their use globally.^11^ Osimertinib, a third-generation epidermal growth factor receptor (EGFR) tyrosine kinase inhibitor (TKI) commonly used in lung cancer with brain metastases, can cost up to $500 000 annually. In comparison, the common neuro-oncologic chemotherapy temozolomide costs anywhere from $2500 to $100 000 for the entirety of treatment.^12^ Similar to temozolomide, many of the other chemotherapies used for gliomas are older medicines without ongoing patent protections. Vorasidenib will likely be priced much higher and closer to the approved IDH inhibitor ivosidenib, which costs >$30 000 USD per month.^13^

These costs greatly impact patients, in terms of both higher health insurance premiums and direct out-of-pocket expenses, such as co-pays and co-insurance.^14^ Research has shown that, on average, US cancer patients spend between $180 and $2600 per month out of pocket on cancer medication, whereas the average in Canada is $15–$400, in Western Europe $4–$609, and in Australia $58–$438.^15^ These high costs do not necessarily translate into quality; recent reports have highlighted that despite the United States spending approximately double what other high-income countries spend on cancer care, cancer mortality rates in the United States are no better than comparable high-income countries that spend less.^16^

## How Medications Are Approved and Priced

### United States

In the United States, the Food and Drug Administration (FDA) approves medications for use after rigorous testing and safety evaluations.^17^ The first stage involves preclinical research, in which the drug is tested using cell cultures, animal testing, and biological assays for both safety and therapeutic purposes. The drug sponsor must submit an Investigational New Drug Application (IND) before conducting human trials. The drug then undergoes Phase 1, Phase 2, and Phase 3 testing before filing for a New Drug Application (NDA). Traditionally, Phase 1 trials involve testing the drug’s safety in a group of healthy volunteers (although this is often not the case for oncology drugs which are typically tested in patients with relapsed/refractory malignancies), Phase 2 tests the drug in people who have the condition the drug is meant to treat and seeks to optimize its dose and treatment schedule, and Phase 3 studies are larger, often randomized, trials that are used to confirm the drug’s effectiveness and side effect profile. These phases are not fixed and may differ based on the disease, especially for rare cancers. After these phases, the sponsor files an NDA with the FDA, which then determines whether to approve the drug for human use. Postmarketing surveillance, also called Phase 4 testing, refers to ongoing monitoring after a drug’s approval to confirm safety and efficacy. Many other countries use a similar system to approve drugs, although, unlike the United States, almost all other high-income countries have a 2-step approval process, where the second step, health technology assessment (HTA), assesses the incremental clinical value of a drug in its disease context and, in many jurisdictions, its cost-effectiveness.

In the United States, there are also multiple incentives to expedite drug development, including breakthrough therapy designation and priority review, and 2 mechanisms for faster approval, fast-track and accelerated approval, which are meant for areas with unmet needs.^18^ Fast track includes rolling reviews and the ability to submit parts of the NDA instead of waiting for all phases to be complete. Accelerated approval is based on improvements in surrogate endpoints, such as a tumor marker or tumor response on imaging, and is often based on single-arm Phase 2 trials.^19^ However, the FDA can withdraw the drug from the market if confirmatory clinical trials do not meet prespecified clinical endpoints.^20^ Recent analysis has shown that a significant portion of cancer drugs that were granted accelerated approval did not improve overall survival (OS) or quality of life in confirmatory trials.^19,21^

In the United States, the approval of a drug and drug pricing are not linked. In contrast to many other countries, the FDA has purview over drug approvals but is specifically legally prohibited from involvement in drug pricing.^22^ Instead, drug pricing in the United States is highly unregulated, largely driven by manufacturers.^23^ The drug manufacturer usually sets the price after analysis of the market size, competition, research and development costs, and therapeutic value. Patents on drugs incentivize drug development but can lead to higher prices during the period of exclusivity, which often lasts much longer than the designated 20 years due to strategic changes in dosing, formulation, or method of administration, and many other legal tactics.^24,25^ This lack of HTA, regulation, or price negotiation may lead to the earlier availability of drugs for patients with comprehensive insurance in the United States, but it can also lead to drug prices that are almost 3.5 times higher than in countries of comparable development.^26^ There are ongoing efforts to reduce drug costs through price negotiation, most notably instituted for the first time by the 2022 Inflation Reduction Act, although this excludes many drugs and only allows negotiation to take effect 9 or 13 years after FDA approval, for small molecules and biologics, respectively (Table 1).^27^

**Table 1.** Approval and Payment of Medications by Country

Country	How Medications Are Approved and Priced
United States	• FDA approves medication after safety testing and evaluation • Drug must pass 3 phases before being able to file a New Drug Application and FDA later decides whether to fully approve drug • Accelerated and fast-track approval options are frequently used in oncology • Drug pricing largely manufacturer-led • Drugs are typically higher priced due to patent-protected monopolies; prices typically fall if generics become available • Inflation Reduction Act will allow some negotiation after 9–13 years • More available for patients with comprehensive (private) insurance
England	• National Institute for Health and Care Excellence (NICE) provides recommendations based on cost-effectiveness to National Health Service (NHS) • Drug companies must submit economic models justifying new drugs and pricing • Cancer Drugs Fund helps to facilitate access for newer cancer treatments
Germany	• Negotiates drug pricing through Federal Association of Sickness Fund • Pricing largely based on reference prices of other drugs • If additional benefits are present, prices can be further negotiated
France	• Negotiates pricing with drug companies • Cannot have price increase at drug release, and price decreases after 5 years • National Authority for Health (HAS) examines benefits through Amélioration du Service Médical Rendu (ASMR) where drugs are scored based on benefits
Italy	• Mostly uses reference pricing by the Italian Medicines Agency • Can further negotiate pricing with drug companies • Committees can also be formed as needed based on drugs with further benefits
Japan	• Pharmaceuticals and Medical Devices Agency (PMDA) manages Japan’s drug approval process • Ministry of Health, Labour, and Welfare (MHLW) set prices of approved medication • Pricing of drugs are regulated every few years
South Africa	• Approved by South African Health Products Regulatory Authority (SAHPRA) • SAHPRA does not consider drug pricing • Government sets price based on single exit price (SEP) • SEP uses a uniform price set by the manufacturer, adjusted for each year due to inflation • The government negotiates pricing for patients with public health insurance
Brazil	• Approved by National Health Surveillance Agency (ANVISA) • Drugs broken down into 2 categories based on effectiveness • Drug Market Regulatory Chamber (CMED) determines pricing in Brazil • Pricing determined by external and internal reference pricing • CMED has maximum prices set and prices are adjusted annually

### Europe

In Europe, drugs can be approved through 3 different regulatory procedures including centralized, decentralized, and national/individual approvals. The centralized approval process is through the European Medicines Agency (EMA), which coordinates and streamlines the drug approval process through the countries in the European Union (EU) and the European Economic Area (EEA). The decentralized procedure refers to a simultaneous application and approval in several countries, but not the entire EU or EEA, while national procedures involve approving a drug in an individual country. Each of these procedures follows a similar process of a drug application to that of the FDA—Phase 1, 2, and 3 testing, followed by formal review—although the bar for regulatory approval and regulatory approaches frequently diverge.^21,28^

Analogous to the FDA’s accelerated approval pathway, the EMA utilizes a pathway called conditional approval for promising drugs with preliminary evidence of effectiveness for unmet medical needs, including in oncology. Drugs are priced through a combination of regulations and negotiation guided by HTAs, unlike in the United States, which utilizes a market-driven approach.^29^ For example, in the United Kingdom, the National Institute for Health and Care Excellence (NICE) evaluates the cost-effectiveness of approved medications and provides recommendations to the National Health Service (NHS). This analysis calculates the cost per quality-adjusted life year (QALY) gained by the new drug. Drug sponsors and companies submit economic models to justify the use of drugs. NICE also consults with stakeholders including patient groups, clinicians, and scientists. NICE then submits a report for guidance to the NHS with a recommendation to either use the drug at its manufacturer-specified price, use the drug but at a lower price, or not use the drug. While these recommendations are not binding, the NHS normally follows the guidance of NICE. For drugs that are expensive but still cost-effective, NICE may negotiate a patient access scheme with the drug manufacturer. Bevacizumab, a monoclonal antibody to vascular endothelial growth factor commonly used in recurrent glioblastoma, was not deemed cost-effective and is therefore not covered by the NHS.^30^ In 2016, to cater to increasingly expensive cancer drugs, the British Government established the Cancer Drugs Fund, which offers interim funding to “provide access to promising new treatments, via managed access arrangement, while further evidence is collected to address clinical uncertainty.”^31^

In Germany, the government negotiates drug prices with drug manufacturers through the Federal Association of Sickness Funds. Usually, drugs are placed into groups with reference prices that determine the maximum amount that can be charged. However, if a drug shows additional benefit compared to other drugs, as evaluated by the Federal Joint Committee, the Federal Association of Sickness Funds can negotiate a higher reimbursement price. This is sometimes seen with drugs for orphan or rare diseases. There is also a 6-month period of market pricing after a drug is approved—the negotiation and benefits analysis occur during this period.

France also negotiates prices with drug companies: after drugs are released, there cannot be any price increases, and the price will decrease after 5 years. The French National Authority for Health (HAS) evaluates a drug’s benefit through the Amélioration du Service Médical Rendu (ASMR). A score is assigned based on the added benefit of the drug compared to existing therapies, and higher scores command higher reimbursements as compared to reference drugs. France keeps overall costs down by mandating that drug manufacturers pay rebates if drug spending exceeds the national pharmaceutical spending cap.^22^ France provides universal health coverage with full coverage for essential medications but only covers a portion of drugs without a significant therapeutic value (SMR). Private coverage options also exist in France.

Italy offers universal healthcare but also has additional private coverage options that cover further services such as medication copayments. Italy also uses reference prices that are set by the Italian Medicines Agency, Agenzia Italiana del Farmaco (AIFA), and negotiates with drug companies to set prices. Some drugs are not reimbursable by public insurance based on HTA review. The law 648/1996 authorizes, following the advice of an “ad hoc” committee, the reimbursement by public health care of innovative drugs that are either commercialized in other nations but not in Italy or are authorized for a different indication. An example is the use of bevacizumab for radiation necrosis (not an officially approved regimen by the Italian Medicines Agency but reimbursable) as compared to that used for the treatment of recurrent glioblastoma (approved and reimbursable).

### Japan

The Pharmaceuticals and Medical Devices Agency (PMDA) oversees Japan’s drug approval process. Similar to other countries, drugs are approved with preclinical testing, clinical trial application, and Phase 1, Phase 2, and Phase 3 testing and then reviewed with the submission of an NDA. Once approved, the Ministry of Health, Labour, and Welfare (MHLW) sets the price of approved medications. Japan offers universal healthcare coverage through the National Health Insurance (NHI), which covers the costs of medications.^32^ To maintain financial stability, especially given Japan’s aging demographics, drug prices are reevaluated every few years. Price cuts and reevaluations are common, and total drug prices have fallen in the past decade as the government seeks to contain costs.^33^ One notable feature of Japan’s regulatory environment is that the PMDA typically requires at least some evidence in support of an NDA to be generated in Japan.

### South Africa

In South Africa, drugs are approved and overseen by the South African Health Products Regulatory Authority (SAHPRA). Once a drug has completed Phase 1, 2, and 3 testing, an application can be submitted to SAHPRA for review. SAHPRA then reviews the application and decides on drug approval. SAHPRA does not take costs into account when deciding on drug approval. However, the government regulates drug prices. South Africa uses a single exit price (SEP), the uniform price at which a manufacturer sells drugs to all pharmacies and clinics in the private sector. This price is adjusted annually based on inflation and other economic factors and takes into account a prescribed mark-up for the manufacturer. For patients with public health insurance, the government negotiates and finds the lowest price for drugs. Access to medications can vary based on one’s health insurance; for patients on public health insurance, there is no cost for medications, but the number of covered drugs is limited. Patients with private insurance pay an additional fee, with improved access to medications.^34^

### Brazil

In Brazil, drugs are approved by the National Health Surveillance Agency (ANVISA). This approval process uses data from Phase 1, 2, and 3 testing and includes a technical and scientific review. ANVISA may facilitate public consultation for certain drug classes to gather opinions from healthcare professionals, patients, and other stakeholders. The Drug Market Regulatory Chamber (CMED) determines drug pricing in Brazil.^35^ Approved drugs are determined to be Category I if they show either greater efficacy, similar efficacy but have the potential to be more cost-effective, or have similar efficacy but with fewer side effects. Drugs that do not meet these criteria are classified as Category II. Pricing is then determined by external and internal reference pricing, with higher prices for Category I medications. CMED sets maximum prices and conducts annual price adjustments. Generic medications are required to be priced below brand-name drugs.

## How Are Drugs Paid for?

As highlighted above, there is much variability in the approval and pricing of drugs globally. In most countries, cost is considered when recommending reimbursement of a drug. Drug pricing is complex and variable, with negotiations between drug manufacturers and governmental bodies. The United States is a notable exception, although new programs are attempting to reduce drug prices.

Even where a drug is approved in a country, it may not be readily available or accessible. An example is the US government insurers, Medicare and Medicaid. Over one-third of the US population is covered by these government plans; Medicare provides coverage for the elderly and some patients with chronic medical conditions, and Medicaid provides coverage for low-income individuals.^36^ Traditionally, anticancer medications were IV infusions and covered under Medicare Part B. Some oral chemotherapies, such as temozolomide, are also covered under Part B. A relatively newer Medicare Part D was created in 2006 to help with oral medication costs, such as newer targeted agents that are not covered by Part B, but these plans still have high out-of-pocket costs,^37^ which often exceed $10 000 USD annually. Of note, Medicare Part D is a voluntary supplementary plan for outpatient prescription benefits and not included in some traditional Medicare plans. One study found that 10% of patients with cancer who are on Medicare spend roughly 60% of their income on cancer treatment.^38^ Indeed, nearly one-third of Medicare Part D beneficiaries without low-income subsidies did not start anticancer drugs they were prescribed; beneficiaries receiving low-income subsidies were twice as likely to obtain their drug.^39^ Recent legislation will impose out-of-pocket caps of $2000 USD per year to help mitigate cost-sharing for patients.^40^ Currently, these caps do not apply to privately insured patients. Medicaid is a joint federal and state program and coverage varies from state to state. Eligibility is income-based, and patients often have no or minimal cost-sharing, but approval of covered medications is slower, and there may be limited availability compared to private insurers. For example, lomustine, the FDA-approved medication for glioblastoma, was removed from Medicaid and Medicare coverage because of lower reimbursement schemes and profit margins for the manufacturer compared to private insurance.^41^ Patients must therefore pay a large out-of-pocket expense unless they qualify for a fee assistance program with the company. The Affordable Care Act (ACA) was passed in 2010 and expanded eligibility for Medicaid coverage up to 138% of the Federal Poverty Level, but requires state input—certain states have not expanded coverage for both political and economic reasons.^42,43^

Many patients in the United States with IDH-mutant gliomas are not eligible for Medicare, given the average age of diagnosis.^44,45^ Instead, commercial and employer-based insurance are the most common forms of coverage. Drug pricing is determined through manufacturer pricing and negotiations with the health insurance company, typically through intermediaries such as pharmacy benefit managers, which are coming under increased scrutiny.^46^ The ACA implemented some out-of-pocket maximums for patients to limit direct cost-sharing, but this can still amount to many thousands of dollars in extra costs for patients.

Outside of the United States, public insurance plans globally reconcile the high costs of anticancer medications with lower reimbursement than in the United States and limiting the availability of or not reimbursing drugs with marginal or questionable benefit. The high costs of medications can place a strain on the system, which can translate into increased premiums (and/or taxes) for residents, or redistribution of other finite healthcare resources. These high prices lead to inequitable and highly variable access to new and expensive medications both between and within countries. Many of the anticancer therapies listed on the World Health Organization’s Essential Medicines List are not covered in national formularies in low- and middle-income countries (LMICs).^47,48^ Furthermore, there is an ongoing debate about whether drugs should be excluded from the list depending on their price, given the potential impact it may have on LMIC health system budgets: on the one hand, including clinically essential but expensive drugs might risk bankrupting health systems, on the other hand, excluding them might falsely increase perceptions of how successful access to high-value drugs is in LMICs.^49,50^ Drug manufacturers make less profit in these countries, so there is limited financial incentive to provide access to medications. Indeed, progress with access to cancer medicines in LMICs remains very slow. To provide resources for these countries, especially given the higher burden of cancer mortality and morbidity, proposals for the Global Fund (or a dedicated Global Cancer Fund) or other international organizations to provide additional resources for cancer drug access have been suggested.^47^ Additionally, LMICs can take various other approaches to securing access to medicines, including voluntary and compulsory licensing.^51,52^

## Considerations of High-Cost Medications—What Makes a Drug Worth it?

Given the increasing costs of anticancer medications, countries, health insurance companies, clinicians, and patients must determine whether to cover a medication. But what makes a drug worth it? Many of the aforementioned countries use formulas and calculations that take into account the efficacy and outcomes of a drug and assign a monetary value to that drug. Some common measurements include life years (LY), which is a metric that measures life expectancy, QALY, which estimates the impact of a drug on overall quality of life and function, and disability-adjusted life year (DALY), the sum of years that are lost from premature mortality and the years due to disability. Incremental cost-effectiveness ratio (ICER) is a metric that compares the difference in cost and outcomes, that is, the extra cost associated with an increase in health effect. These measures are compared to the overall cost of the drug and often compared to a cost-effectiveness threshold, which helps to enable comparisons between therapeutic areas. Although various high-income countries use different thresholds when evaluating drugs, the WHO cost-effectiveness threshold is equal to 3 times the national annual GDP per capita of a country.^29,53,54^

An example from thoracic oncology is osimertinib. Osimertinib is now considered an appropriate first-line treatment for patients with EGFR-mutated non-small cell lung cancer, based on its efficacy and tolerability. However, multiple studies comparing the cost-effectiveness of osimertinib to other EGFR-targeted therapies and to traditional chemoimmunotherapy have not found it to be cost-effective. Cost-effectiveness analysis from the landmark FLAURA trial (osimertinib as first-line therapy for untreated EGFR-mutant NSCLC) showed that the incremental cost was $225 000 per QALY in the United States and $172 000 USD per QALY in Brazil, which did not meet the WHO cost-effectiveness threshold.^55^ Another study showed that first-line therapy and second-line use after another EGFR therapy was not cost-effective in either the United States or China.^56^ Cost-effectiveness does not always correlate with efficacy—based on efficacy determined by randomized clinical trials, osimertinib is often a clinically superior option when compared to standard chemoimmunotherapy or older EGFR therapies. However, the cost of the medication puts a strain on both payers and patients.

### Vorasidenib

A similar analysis could also be completed on vorasidenib once it is priced, and additional outcomes data—particularly regarding quality of life and functional outcomes—become available. Health economists will calculate an ICER and cost-benefit for vorasidenib. Important factors to consider are whether the increase in progression-free survival (PFS) and the delay in chemotherapy and radiation offer a true quality of life benefit, and/or save money. This will be quantified for both QALYs and DALYs. Given the unique nature of neuro-oncology patients, with potentially subtle deficits caused by both the tumor and therapy, it may be challenging to accurately quantify the QALY benefit of delaying radiation and chemotherapy, stressing the importance of collection and rapid publication of robust quality-of-life data. The reporting of these data is the bottleneck for a comprehensive cost-effectiveness analysis; once released, an analysis can be completed relatively quickly. Another consideration is whether treatment with vorasidenib has an OS benefit, which may take years to determine. For comparison, the pivotal RTOG 9802 trial, which compared procarbazine, lomustine, and vincristine after radiation therapy to radiation therapy alone, initially reported longer PFS but not OS.^57^ The long-term results that showed an OS benefit were presented over a decade after the last patient was enrolled in the study.^58,59^

On the individual level, it may also take months to years to determine if the medication is truly working for a patient, due to the indolent nature of the disease. This may lead to patients who derive minimal or no benefit but will stay on an expensive medication for a significant length of time. Clinicians will need to be conscious about progression, especially treatment after progression, and use clear guidelines such as the Response Assessment in Neuro-Oncology for low-grade gliomas (RANO-LGG) to evaluate radiographic response or the Neurologic Assessment in Neuro-Oncology (NANO) scale for clinical response.^60,61^

## Financial Toxicity and Treatment Adherence

Cancer medications, especially long-term cancer medications, can place extreme financial burdens on cancer patients. One term for this is financial toxicity, which is a combination of the objective financial burden and the subjective financial distress that patients face because of the cost of their treatment.^62^ Financial toxicity is widespread, particularly in countries that require significant cost-sharing from patients. In the United States, half of adults have difficulty paying for any medical costs, with over a quarter having difficulty paying for prescription drugs.^63^ Twenty percent of patients did not fill a prescription medication due to cost.^39,63^

Financial toxicity can cause physiological and psychological symptoms and distress. Cancer patients with financial toxicity are more likely to exhibit anxiety, depression, decreased coping and stress management, and decline in overall well-being.^3,62,64,65^ Additionally, financial toxicity and high out-of-pocket costs leave patients with less money for housing, transportation, food, and other essential needs. This is especially important as many patients with cancer face unemployment and decreased earning potential compared to prediagnosis.^66^ This can lead to a vicious cycle in which patients are faced with a diagnosis requiring significant additional costs, lose their jobs, or are unable to work because of their cancer diagnosis (which, in a system such as the US system where health insurance is often tied to employment, can lead to loss of health insurance also), and use savings and exhaust all other sources of income.^67^ This results in medical debt and prioritizing medical care at the expense of essential needs.

One study found that 46% of patients experiencing financial toxicity decreased spending on clothes and food, and 68% decreased spending on leisure activities.^68^ Twenty percent of patients took less of the prescribed medication to extend its use, and a similar percentage did not fill their prescription at all.^68^ Prescription abandonment, the situation in which a prescription is written but never filled or dispensed, is also higher with expensive medications. This has a profound impact on health equity, as patients with lower socioeconomic status are more likely to experience financial toxicity and subsequently have lower rates of treatment adherence.^69^

This is extremely pertinent for patients with low-grade gliomas who can experience neuropsychiatric, neurocognitive, motor or sensory symptoms, aphasias, or epilepsy. All of these symptoms can interfere with professional and work activities, with increased symptom burden correlating with a lower likelihood of returning to work.^70^ A study from Sweden found that just 52% and 63% of patients returned to work at 1 and 2 years after surgery for a low-grade glioma, respectively.^71^ This has many implications for patients whose insurance is tied to employment. If patients cannot return to work, they may lose their insurance and are not eligible for some safety net programs. Unlike Grade 3 or 4 tumors, which qualify for Social Security disability benefits in the United States, low-grade tumors do not automatically qualify for additional benefits.^72^ This underinsurance may place additional stress on patients and also make it difficult to obtain high-cost medications such as vorasidenib. The link between employment and insurance also leads to patients and caregivers feeling unable to leave a job for fear of losing health insurance benefits, which is known as “job lock.” One study found that 1 in 3 cancer survivors or spouses/partners in the United States experience job lock.^73^ This can lead to a difficult situation for patients with low-grade gliomas; they may feel pressure to stay or return to a job for employment benefits, but neurologic symptoms make it difficult to continue working. Patients with low-grade gliomas are at very high risk of experiencing financial toxicity, both through the disease process and treatment and from symptoms of the disease that cause additional financial burden and difficulties with professional activities (Table 2).

**Table 2.** Financial Toxicity and Potential Quality of Life Implications for Patients With Low-Grade Gliomas

Category	Item	Quality of Life Impact	Specific Obstacles
Direct medical costs	Surgery	Physical recovery, potential complications	Long hospital stays, risk of complications
	Radiation therapy	Fatigue, cognitive effects	Frequent treatments, travel needs
	Chemotherapy	Side effects like nausea, fatigue	Managing medications, ongoing treatments
	Medications	Managing side effects, adherence	Regular monitoring
Indirect costs	Lost income	Financial stress	Inability to work, reliance on benefits
	Travel expenses	Exhaustion, time away from home	Frequent travel for treatment
	Childcare	Stress of managing responsibilities	Additional childcare needs
Long-term financial burdens	Rehabilitation	Physical and cognitive rehabilitation	Long-term therapy sessions
	Ongoing medical care	Regular follow-ups	Continuous monitoring and appointments
	Home care	Assistance with daily activities	Home modifications, hiring aides

### Vorasidenib

Based on evidence from other cancers and targeted therapies, vorasidenib will likely result in substantial financial toxicity and limited patient access due to its price. Treatment adherence, the extent to which a patient takes a medication as prescribed, will vary depending on the cost and availability of the medication. As vorasidenib will be dosed daily, this will be important to monitor. If co-pays and costs are high for patients, we can extrapolate that some patients may miss doses to make the prescription last longer or abandon prescriptions altogether. However, the high costs of medications may to some extent be mitigated by economic productivity and work. For example, if vorasidenib delays chemoradiation, it may result in a greater percentage of patients returning to work after the diagnosis. It may also result in more patients returning to full-time work rather than part-time work, which may increase personal income. This will need to be studied closely as the introduction of costly, daily medications for patients with low-grade gliomas can exacerbate financial toxicity in this already vulnerable group.

While the initial costs of vorasidenib may be higher as the manufacturer recoups expenses from research and development and capitalizes on the results of the INDIGO trial, there are additional factors that may drive the price down. There are several ongoing trials evaluating different IDH inhibitors—if these medications are efficacious and improved, then theoretically this could drive the cost down due to competition, although this phenomenon has typically not been observed in pharmaceutical markets.^2,74^ There must also be an intentional approach to health policy that ensures any cost savings are passed down to patients, which has not always been the case.^75^ Additionally, postmarketing surveillance and de-escalation studies may indicate trends on the optimal duration and dosing of treatment, similar to other cancers.^76^ As with other medications, governments, health insurance companies, and intermediaries should negotiate with the manufacturer to find a price that makes the medication widely available.

## Conclusion

Drug pricing and approval differ throughout the world. Many countries use cost-effectiveness analysis in the HTA process to decide which therapies offer the greatest value for both patients and taxpayers. This helps to curtail costs but may limit the availability of marginal or expensive medications. Some countries, such as the United States, let drug manufacturers set prices and subsequently pay substantially more for prescription medications but have widespread access to medications for patients with high-quality health insurance. Pricing, regulations, and the implications of pricing will become increasingly pertinent to our field and patients as new treatments are developed. Vorasidenib has the potential to delay chemotherapy and radiation therapy in patients with IDH-mutated gliomas. This may improve the quality of life for patients, although this has yet to be demonstrated in a clinical trial. However, the cost-effectiveness of this putative benefit will need to be calculated. Additionally, the cost of vorasidenib may be prohibitive for much of the world’s population. This is an exciting time for patients with IDH-mutant tumors, but substantial uncertainties about drug access and. availability remain.

## References

[1] Mellinghoff IK , Lu M, Wen PY, et al Vorasidenib and ivosidenib in IDH1-mutant low-grade glioma: a randomized, perioperative phase 1 trial. Nat Med. 2023;29(3):615–622. 36823302 10.1038/s41591-022-02141-2 PMC10313524

[2] Rudà R , Horbinski C, van den Bent M, Preusser M, Soffietti R. IDH inhibition in gliomas: from preclinical models to clinical trials. Nat Rev Neurol. 2024;20(7):395–407. 38760442 10.1038/s41582-024-00967-7

[3] Tran G , Zafar SY. Financial toxicity and implications for cancer care in the era of molecular and immune therapies. Ann Transl Med. 2018;6(9):166. 29911114 10.21037/atm.2018.03.28 PMC5985271

[4] Yousuf Zafar S. Financial toxicity of cancer care: it’s time to intervene. J Natl Cancer Inst. 2016;108(5):djv370. 26657334 10.1093/jnci/djv370

[5] Rome BN , Egilman AC, Kesselheim AS. Trends in prescription drug launch prices, 2008-2021. JAMA. 2022;327(21):2145–2147. 35670795 10.1001/jama.2022.5542 PMC9175077

[6] U.S. Health Care from a Global Perspective, 2022. *Accelerating Spending, Worsening Outcomes*. (2023, January 31). Commonwealth Fund.

[7] Rajkumar S. The high cost of prescription drugs: causes and solutions. Blood Cancer J. 2020;10(6):71. 32576816 10.1038/s41408-020-0338-x PMC7311400

[8] Ledley FD , McCoy SS, Vaughan G, Cleary EG. Profitability of large pharmaceutical companies compared with other large public companies. JAMA. 2020;323(9):834–843. 32125401 10.1001/jama.2020.0442 PMC7054843

[9] Angelis A , Polyakov R, Wouters OJ, Torreele E, McKee M. High drug prices are not justified by industry’s spending on research and development. BMJ. 2023;380(8371):e071710. 36792119 10.1136/bmj-2022-071710

[10] Tay-Teo K , Ilbawi A, Hill SR. Comparison of sales income and research and development costs for FDA-approved cancer drugs sold by originator drug companies. JAMA Netw Open. 2019;2(1):e186875. 30644967 10.1001/jamanetworkopen.2018.6875 PMC6324319

[11] Cliff ERS , Kelkar AH, Russler-Germain DA, et al High cost of chimeric antigen receptor T-cells: challenges and solutions. Am Soc Clin Oncol Educ Book. 2023;43(43):e397912. 37433102 10.1200/EDBK_397912

[12] Gupta N , Prinja S, Patil V, Bahuguna P. Cost-effectiveness of temozolamide for treatment of glioblastoma multiforme in India. JCO Glob Oncol. 2021;7(7):108–117. 33449801 10.1200/GO.20.00288 PMC8081547

[13] Voranigo, Tibsovo. Merative Micromedex Red Book. Merative Micromedex. Merative; 2024. Accessed September 3, 2024.

[14] Hwang TJ , Qin X, Keating NL, Huskamp HA, Dusetzina SB. Assessment of out-of-pocket costs with rebate pass-through for brand-name cancer drugs under Medicare Part D. JAMA Oncol. 2022;8(1):155–156. 34762097 10.1001/jamaoncol.2021.5433 PMC8587215

[15] Iragorri N , de Oliveira C, Fitzgerald N, Essue B. The out-of-pocket cost burden of cancer care—a systematic literature review. Curr Oncol. 2021;28(2):1216–1248. 33804288 10.3390/curroncol28020117 PMC8025828

[16] Blumenthal D , Gumas ED, Shah A, Gunja MZ, Williams II RD. Mirror, Mirror 2024: A Portrait of the Failing U.S. Health System. Commonwealth Fund.

[17] Center for Drug Evaluation and Research. Development & Approval Process | Drugs. FDA. July 18, 2023. https://www.fda.gov/drugs/development-approval-process-drugs. Accessed June 19, 2024.

[18] Hwang TJ , Franklin JM, Chen CT, et al Efficacy, safety, and regulatory approval of food and drug administration-designated breakthrough and nonbreakthrough cancer medicines. J Clin Oncol. 2018;36(18):1805–1812. 29688832 10.1200/JCO.2017.77.1592

[19] Liu ITT , Kesselheim AS, Cliff ERS. Clinical benefit and regulatory outcomes of cancer drugs receiving accelerated approval. JAMA. 2024;331(17):1471–1479. 38583175 10.1001/jama.2024.2396 PMC11000139

[20] Gyawali B , Rome BN, Kesselheim AS. Regulatory and clinical consequences of negative confirmatory trials of accelerated approval cancer drugs: retrospective observational study. BMJ. 2021;374(8305):n1959. 34497044 10.1136/bmj.n1959 PMC8424519

[21] Koole SN , Huisman AH, Timmers L, et al Lessons learned from postmarketing withdrawals of expedited approvals for oncology drug indications. Lancet Oncol. 2024;25(3):e126–e135. 38423058 10.1016/S1470-2045(23)00592-2

[22] Raimond VC , Feldman WB, Rome BN, Kesselheim AS. Why France spends less than the United States on drugs: a comparative study of drug pricing and pricing regulation. Milbank Q. 2021;99(1):240–272. 33751664 10.1111/1468-0009.12507 PMC7984670

[23] Kesselheim AS , Avorn J, Sarpatwari A. The high cost of prescription drugs in the United States origins and prospects for reform. JAMA. 2016;316(8):858–871. 27552619 10.1001/jama.2016.11237

[24] Vokinger KN , Kesselheim AS, Avorn J, Sarpatwari A. Strategies that delay market entry of generic drugs. JAMA Intern Med. 2017;177(11):1665–1669. 28975217 10.1001/jamainternmed.2017.4650

[25] Horrow C , Gabriele SME, Tu SS, Sarpatwari A, Kesselheim AS. Patent portfolios protecting 10 top-selling prescription drugs. JAMA Intern Med. 2024;184(7):e240836. 10.1001/jamainternmed.2024.0836 PMC11091822 38739386

[26] Ginsburg P , Lieberman SM. Government Regulated or Negotiated Drug Prices: Key Design Considerations. Brookings. https://www.brookings.edu/articles/government-regulated-or-negotiated-drug-prices-key-design-considerations/. Accessed May 2, 2024.

[27] Sullivan SD. Medicare drug price negotiation in the United States: implications and unanswered questions. Value Health. 2023;26(3):394–399. 36503034 10.1016/j.jval.2022.11.015

[28] Cramer A , Sørup FKH, Christensen HR, Petersen TS, Karstoft K. Withdrawn accelerated approvals for cancer indications in the USA: what is the marketing authorisation status in the EU? Lancet Oncol. 2023;24(9):e385–e394. 37657479 10.1016/S1470-2045(23)00357-1

[29] Rand LZ , Kesselheim AS. An international review of health technology assessment approaches to prescription drugs and their ethical principles. J Law Med Ethics. 2020;48(3):583–594. 33021189 10.1177/1073110520958885

[30] NICE. Bevacizumab for Treating Recurrent Glioblastoma [ID978]. https://www.nice.org.uk/guidance/discontinued/gid-ta10149. Accessed May 6, 2024.

[31] NHS England. Cancer Drugs Fund. https://www.england.nhs.uk/cancer/cdf/. Accessed June 9, 2024.

[32] Tikkanen R , Osborn R, Mossialos E, Djordjevic A, Wharton GA. Japan. June 5, 2020. https://www.commonwealthfund.org/international-health-policy-center/countries/japan. Accessed May 2, 2024.

[33] Lin C. Japan’s FY 2024 Pricing Reform Expected to Favour New Listed Innovative Drugs. Pharmaceutical Technology. February 15, 2024. https://www.pharmaceutical-technology.com/analyst-comment/japan-fy-2024-pricing-reform-expected-to-favour-new-listed-innovative-drugs/. Accessed May 2, 2024.

[34] Gray A , Riddin J, Jugathpal J. Health care and pharmacy practice in South Africa. Can J Hosp Pharm. 2016;69(1):36–41. 26985087 10.4212/cjhp.v69i1.1521 PMC4777579

[35] Ivama-Brummell AM , Marciniuk FL, Wagner AK, et al Marketing authorisation and pricing of FDA-approved cancer drugs in Brazil: a retrospective analysis. Lancet Reg Health Am. 2023;22:100506. 37235087 10.1016/j.lana.2023.100506 PMC10206192

[36] Keisler-Starkey K , Bunch LN, Lindstrom RA. Health Insurance Coverage in the United States: 2022. United States Census Bureau. https://www.census.gov/library/publications/2023/demo/p60-281.html. Accessed May 12, 2024.

[37] Kyle MA , Dusetzina SB, Keating NL. Evaluation of trends in oncology drug spending in Medicare, 2016 to 2020. JAMA Netw Open. 2022;5(7):e2221468. 35816318 10.1001/jamanetworkopen.2022.21468 PMC9280395

[38] Narang AK , Nicholas LH. Out-of-pocket spending and financial burden among Medicare beneficiaries with cancer. JAMA Oncol. 2017;3(6):757–765. 27893028 10.1001/jamaoncol.2016.4865 PMC5441971

[39] Dusetzina SB , Huskamp HA, Rothman RL, et al Many Medicare beneficiaries do not fill high-price specialty drug prescriptions. Health Aff (Millwood). 2022;41(4):487–496. 35377748 10.1377/hlthaff.2021.01742

[40] Cubanski J , Published TN. The New Help for Medicare Beneficiaries with High Drug Costs That Few Seem to Know About. KFF. December 12, 2023. https://www.kff.org/policy-watch/the-new-help-for-medicare-beneficiaries-with-high-drug-costs-that-few-seem-to-know-about/. Accessed May 12, 2024.

[41] Michaelson NM , Watsula A, Bakare-Okpala A, et al Disparities in neuro-oncology. Curr Neurol Neurosci Rep. 2023;23(12):815–825. 37889427 10.1007/s11910-023-01314-x

[42] Haeder SF , Weimer DL. You can’t make me do it: state implementation of insurance exchanges under the Affordable Care Act. Public Adm Rev. 2013;73(s1):S34–S47.

[43] Status of State Medicaid Expansion Decisions: Interactive Map. KFF. May 8, 2024. https://www.kff.org/affordable-care-act/issue-brief/status-of-state-medicaid-expansion-decisions-interactive-map/. Accessed August 12, 2024.

[44] Ostrom QT , Price M, Neff C, et al CBTRUS statistical report: primary brain and other central nervous system tumors diagnosed in the United States in 2015-2019. Neuro Oncol. 2022;24(Suppl 5):v1–v95. 36196752 10.1093/neuonc/noac202 PMC9533228

[45] Claus EB , Walsh KM, Wiencke J, et al Survival and low grade glioma: the emergence of genetic information. Neurosurg Focus. 2015;38(1):E6. 10.3171/2014.10.FOCUS12367 PMC4361022 25552286

[46] Berndt ER , McGuire T, Newhouse JP. A primer on the economics of prescription pharmaceutical pricing in health insurance markets. Forum Health Econ Policy. 2011;14(1):1–39.

[47] Cortes J , Perez-García JM, Llombart-Cussac A, et al Enhancing global access to cancer medicines. CA Cancer J Clin. 2020;70(2):105–124. 32068901 10.3322/caac.21597

[48] Ruff P , Al-Sukhun S, Blanchard C, Shulman LN. Access to cancer therapeutics in low- and middle-income countries. Am Soc Clin Oncol Educ Book. 2016;36(36):58–65. 10.1200/EDBK_155975 27249686

[49] Hwang TJ , Kesselheim AS, Vokinger KN. Reforming the World Health Organization’s Essential Medicines List: essential but unaffordable. JAMA. 2022;328(18):1807–1808. 36279114 10.1001/jama.2022.19459

[50] Fojo T. The challenges of selecting cancer medicines for the WHO Essential Medicines List with the elephant in the room: a path forward. Semin Oncol. 2022;49(6):427–428. 36754713 10.1053/j.seminoncol.2023.01.005

[51] Bognar CLFB , Bychkovsky BL, Lopes GL Jr. Compulsory licenses for cancer drugs: does circumventing patent rights improve access to oncology medications? J Global Oncol. 2016;2(5):292–301. 10.1200/JGO.2016.005363 PMC5493268 28717715

[52] Dare AJ , Bayle A, Hatoqai A, et al Ensuring global access to cancer medicines: a generational call to action. Cancer Discov. 2023;13(2):269–274. 36734325 10.1158/2159-8290.CD-22-1372

[53] Rand LZ , Raymakers A, Rome BN. Congress’ misguided plan to ban QALYs. JAMA. 2023;329(24):2125–2126. 37289466 10.1001/jama.2023.8695

[54] Marseille E , Larson B, Kazi DS, Kahn JG, Rosen S. Thresholds for the cost–effectiveness of interventions: alternative approaches. Bull World Health Organ. 2015;93(2):118–124. 25883405 10.2471/BLT.14.138206 PMC4339959

[55] Aguiar PN, Jr, Haaland B, Park W, et al Cost-effectiveness of osimertinib in the first-line treatment of patients with EGFR-mutated advanced non–small cell lung cancer. JAMA Oncol. 2018;4(8):1080–1084. 29852038 10.1001/jamaoncol.2018.1395 PMC6143050

[56] Wu B , Gu X, Zhang Q, Xie F. Cost-effectiveness of osimertinib in treating newly diagnosed, advanced EGFR-mutation-positive non-small cell lung cancer. Oncologist. 2019;24(3):349–357. 30257889 10.1634/theoncologist.2018-0150 PMC6519771

[57] Shaw EG , Wang M, Coons SW, et al Randomized trial of radiation therapy plus procarbazine, lomustine, and vincristine chemotherapy for supratentorial adult low-grade glioma: initial results of RTOG 9802. J Clin Oncol. 2012;30(25):3065–3070. 22851558 10.1200/JCO.2011.35.8598 PMC3732006

[58] van den Bent MJ. Practice changing mature results of RTOG study 9802: another positive PCV trial makes adjuvant chemotherapy part of standard of care in low-grade glioma. Neuro Oncol. 2014;16(12):1570–1574. 25355680 10.1093/neuonc/nou297 PMC4232091

[59] Buckner JC , Shaw EG, Pugh SL, et al Radiation plus procarbazine, CCNU, and vincristine in low-grade glioma. N Engl J Med. 2016;374(14):1344–1355. 27050206 10.1056/NEJMoa1500925 PMC5170873

[60] Wen PY , van den Bent M, Youssef G, et al RANO 2.0: update to the response assessment in neuro-oncology criteria for high- and low-grade gliomas in adults. J Clin Oncol. 2023;41(33):5187–5199. 37774317 10.1200/JCO.23.01059 PMC10860967

[61] Nayak L , DeAngelis LM, Brandes AA, et al The Neurologic Assessment in Neuro-Oncology (NANO) scale: a tool to assess neurologic function for integration into the Response Assessment in Neuro-Oncology (RANO) criteria. Neuro Oncol. 2017;19(5):625–635. 28453751 10.1093/neuonc/nox029 PMC5464449

[62] Carrera PM , Kantarjian HM, Blinder VS. The financial burden and distress of patients with cancer: understanding and stepping-up action on the financial toxicity of cancer treatment. CA Cancer J Clin. 2018;68(2):153–165. 29338071 10.3322/caac.21443 PMC6652174

[63] Lopes L , Montero A, Presiado M, Published LH. Americans’ Challenges with Health Care Costs. KFF. March 1, 2024. https://www.kff.org/health-costs/issue-brief/americans-challenges-with-health-care-costs/. Accessed May 11, 2024.

[64] Pisu M , Martin MY. Financial toxicity: a common problem affecting patient care and health. Nat Rev Dis Primers. 2022;8(1):7. 35145106 10.1038/s41572-022-00341-1 PMC9731797

[65] Thom B , Benedict C. The impact of financial toxicity on psychological well-being, coping self-efficacy, and cost-coping behaviors in young adults with cancer. J Adolesc Young Adult Oncol. 2019;8(3):236–242. 30817217 10.1089/jayao.2018.0143 PMC6588118

[66] Blinder VS , Gany FM. Impact of cancer on employment. J Clin Oncol. 2020;38(4):302–309. 31804857 10.1200/JCO.19.01856 PMC6992498

[67] Yabroff KR , Doran JF, Zhao J, et al Cancer diagnosis and treatment in working-age adults: implications for employment, health insurance coverage, and financial hardship in the United States. CA Cancer J Clin. 2024;74(4):341–358. 38652221 10.3322/caac.21837

[68] Zafar SY , Peppercorn JM, Schrag D, et al The financial toxicity of cancer treatment: a pilot study assessing out-of-pocket expenses and the insured cancer patient’s experience. Oncologist. 2013;18(4):381–390. 23442307 10.1634/theoncologist.2012-0279 PMC3639525

[69] Wong YN , Egleston BL, Sachdeva K, et al Cancer patients’ trade-offs among efficacy, toxicity, and out-of-pocket cost in the curative and noncurative setting. Med Care. 2013;51(9):838–845. 23872905 10.1097/MLR.0b013e31829faffd PMC3857689

[70] Leeper HE , Vera E, Christ A, et al Association of employment status with symptom burden and health-related quality of life in people living with primary CNS tumors. Neurology. 2023;100(16):e1723–e1736. 36754634 10.1212/WNL.0000000000207082 PMC10115506

[71] Rydén I , Carstam L, Gulati S, et al Return to work following diagnosis of low-grade glioma: a nationwide matched cohort study. Neurology. 2020;95(7):e856–e866. 32540938 10.1212/WNL.0000000000009982 PMC7605502

[72] Yogendran L , Rudolf M, Yeannakis D, Fuchs K, Schiff D. Navigating disability insurance in the American healthcare system for the low-grade glioma patient. Neuro-Oncol Pract. 2023;10(1):5–12. 10.1093/nop/npac076 PMC9837773 36659964

[73] Kent EE , de Moor JS, Zhao J, et al Staying at one’s job to maintain employer-based health insurance among cancer survivors and their spouses/partners. JAMA Oncol. 2020;6(6):929–932. 32324208 10.1001/jamaoncol.2020.0742 PMC7180727

[74] Sarpatwari A , DiBello J, Zakarian M, Najafzadeh M, Kesselheim AS. Competition and price among brand-name drugs in the same class: a systematic review of the evidence. PLoS Med. 2019;16(7):e1002872. 31361747 10.1371/journal.pmed.1002872 PMC6667132

[75] Feng K , Russo M, Maini L, Kesselheim AS, Rome BN. Patient out-of-pocket costs for biologic drugs after biosimilar competition. JAMA Health Forum. 2024;5(3):e235429. 38551589 10.1001/jamahealthforum.2023.5429 PMC10980968

[76] Gnant M , Fitzal F, Rinnerthaler G, et al; Austrian Breast and Colorectal Cancer Study Group. Duration of adjuvant aromatase-inhibitor therapy in postmenopausal breast cancer. N Engl J Med. 2021;385(5):395–405. 34320285 10.1056/NEJMoa2104162

